# Prediction of Fruity Aroma Intensity and Defect Presence in Virgin Olive Oil Using an Electronic Nose

**DOI:** 10.3390/s21072298

**Published:** 2021-03-25

**Authors:** Pablo Cano Marchal, Chiara Sanmartin, Silvia Satorres Martínez, Juan Gómez Ortega, Fabio Mencarelli, Javier Gámez García

**Affiliations:** 1 Robotics, Automation and Computer Vision Group, University of Jaén, 23071 Jaén, Spain; satorres@ujaen.es (S.S.M.); juango@ujaen.es (J.G.O.); jggarcia@ujaen.es (J.G.G.); 2 Department of Agriculture, Food and Environment, University of Pisa, 56126 Pisa, Italy; chiara.sanmartin@unipi.it (C.S.); mencarelli@unipi.it (F.M.)

**Keywords:** virgin olive oil, quality, electronic nose

## Abstract

The organoleptic profile of a Virgin Olive Oil is a key quality parameter that is currently obtained by human sensory panels. The development of an instrumental technique capable of providing information about this profile quickly and online is of great interest. This work employed a general purpose e-nose, in lab conditions, to predict the level of fruity aroma and the presence of defects in Virgin Olive Oils. The raw data provided by the e-nose were used to extract a set of features that fed a regressor to predict the level of fruity aroma and a classifier to detect the presence of defects. The results obtained were a mean validation error of 0.5 units for the prediction of fruity aroma using lasso regression; and 88% accuracy for the defect detection using logistic regression. Finally, the identification of two out of ten specific sensors of the e-nose that can provide successful results paves the way to the design of low-cost specific electronic noses for this application.

## 1. Introduction

A common feature of the Mediterranean diet, which is a dietary pattern associated with longevity, improvement of life quality, and a reduction of the incidence of many chronic pathologies (i.e., cardiovascular diseases, cerebrovascular diseases, diabetes mellitus, metabolic syndrome, certain cancers, and neurodegenerative diseases), is the high consumption of olives and olive oil as the primary source of dietary fat [1,2,3].

Olive oil quality is deeply conditioned by extraction techniques, thereafter virgin olive oils are designated also as “oils obtained from the fruit of the olive tree solely by mechanical or other physical means under conditions, particularly thermal conditions that do not lead to alterations in the oil, and which have not undergone any treatment other than washing, decantation, centrifugation and filtration”. These oils are ready for direct human consumption (EC Reg ECC 2568/1991 as amended). European legislation provides three main merceological classes of olive oil, based on physical, chemical, and sensory characteristics: extra virgin (EVOO), virgin (VOO), and lampante olive oil (LOO). The olive oil features are strictly regulated by EU, which also establishes methods for their analysis (Reg (EEC) 2568/91, EC Reg 640/08) [4]. When EVOO is extracted from fresh and healthy olive fruits, it is properly processed and adequately stored [5,6]; it exhibits an organoleptical profile characterized by a unique fruity aroma and taste, with no defects (e.g., fusty, musty, winey, metallic, rancid).

Its volatile constituents are mainly C6 compounds (aldehydes, ketones, alcohols, and esters in particular), synthesized by means of the lipoxygenase metabolic pathway and responsible for EVOO green scents [7]. EVOO’s fragrant and delicate flavor is ascribable to fruity sensation, which is a reminiscence of the healthy olive fruit perfectly ripe, and to scents of cut grass, olive leaf, flowery scents, green fruits (e.g., apple, banana, almond) or vegetables (e.g., tomato, artichoke) [8,9].

Indeed, its peculiar taste is characterized by more or less intense bitterness and pungency notes, which are positive attributes depending on the content of bioactive compounds strictly related to important health benefits, such as phenols [10]. The EVOO sensory profile is strongly related to its quality, so there are other lower-quality olive oils, such as VOO, which is also fruity but has a few minor defects deriving from incorrect extraction or storage processes, and LOO, which exhibits an unpleasant smell and is non-edible [11].

According to the European legislation, the sensory analysis of virgin olive oils must be carried out by both smell and taste assessments by means of a panel test, constituted by a group of trained and selected assessors (EEC 2568/91; EC 640/08). Thus far, there is no methodology able to suitably replace human sensory panels, essentially because taste and olfactory properties are formed by a plethora of different compounds in varying concentrations, which deliver the holistic sensory impression [12,13,14]. Sensory assessment, however, needs many resources and time, as well as specialized panellists, which are not always at the disposal of small/medium-sized enterprises and cooperative societies, and should not be used for routine operations [13,15].

In this context, there is a need for the development of accurate instrumental techniques capable of performing measurements in real-time and generating the same information as a panel, in a reproducible and stable way, in order to achieve the correct classification of VOOs rapidly and efficiently [16]. Several analytical methods have been proposed in different studies carried out in the last decade, many including characterizations of the volatile markers for each sensory characteristics [17], and there have been recent efforts devoted to the quality testing of different types of oils using electronic noses [18,19,20]. The development of easy-to-use electronic noses, capable of being installed directly in the process line, appears extremely appealing in this field.

The aim of this work is to employ the electronic nose to develop an automatic online analysis system for olive oils’ batches sensory classification immediately after the olive oil extraction process. The acquired knowledge could be applied to evaluate the fruity aroma and defect level of the olive oil batches at the end of the virgin olive oil production process and classify them, in order to avoid misclassification and to prevent the mixing low quality oil in the same tank with high quality oil. In this work, we analyze all the VOO produced by a company in a season in lab conditions, and assess its quality by means of predicting the value of the fruity aroma attribute and the existence of defects.

The rest of the paper is organized as follows: the next section provides a brief overview of relevant previous works, while Section 3 details the Materials and Methods employed to obtain the results discussed in Section 4. The paper ends with the conclusions presented in Section 5.

## 2. Related Works

The sensors usually denominated electronic noses are devices that employ one or more gas transducers that receive a flow of air extracted from the headspace of the samples and modify their electric impedance in the presence of different families of volatile molecular compounds, so that they provide an electric signal that depends on the presence or absence of these compounds [21].

The major applications in food products for this type of sensors are quality control and food safety [22,23], authenticity control [24], and detection of contaminants [25]. This way, in the literature, there are several reports that devote their attention to the application of e-noses to fruits and vegetables in order to identify and select cultivars, to discriminate among different ripening stages, to predict the optimal harvest date, to monitor shelf life [26] or to manage the post-harvest processes and to evaluate the occurrence of defects [16]. This technology has also been employed to evaluate the quality of fish [27,28], meat [29] and processed vegetables [25,30,31,32].

In addition, according to [23], e-noses find useful applications in several issues pertaining to classification, determination of botanical and geographical origin, detection of adulterations and evaluation of the degree of oxidation of edible oils [33]. For olive oil in particular, this technology has been proven useful for determining the geographical origin of the oil, detecting adulterations and quality determining the spoilage due to external factors, such as temperature, storage time, air composition in storage tanks, etc. [23].

Other recent studies of the application of this technology to olive oil [34,35] show the different responses provided by the sensors when confronted with different olive oil samples adulterated with other vegetal oils. Ref. [36] show the possibility of classifying virgin olive oil versus other chemically treated olive oils using only a single sensor, with a success rate of 91%. Other works, such as [37], show the use of e-nose to detect the quality loss over time of virgin olive oils stored at different temperatures, with a success rate close to 94%. In turn, ref. [38] used an e-nose to classify olive oils obtained from different cultivars, yielding a 82% accuracy in their predictions. Finally, ref. [39] demonstrated the potential of using this type of technology directly on intact olives to predict whether the oil to be produced from them would be virgin or lampante, with an accuracy of 90%.

The novelty of the work presented in this paper resides on the prediction of the intensity of fruity aroma and the detection of defect, which can be employed to classify olive oils prior to their storage.

## 3. Materials and Methods

### 3.1. Olive Oil Samples

Forty-two olive oil samples, produced from *Olea europeae* fruits belonging to typical Spanish cultivars (mainly cv. Picual) and harvested during the 2018/2019 crop season from different growers of Jaén province (Spain), were provided by a local Olive Oil Cooperative (Picualia mill, Bailen—Jaén).

Each sample was divided in representative aliquots and stored in dark glass 125 mL containers with the aim to immediately evaluate its volatile emission by electronic-nose and to be delivered to the accredited CM Europe laboratory for the sensory and chemical analysis.

### 3.2. Chemical and Sensory Analysis of Oil

Each chemical determination was performed in triplicate, following the official methods—acidity index (AI), peroxide value (PV), K232, K270, and ΔK according to EU Reg 2568/1991. The sensory characterization followed the method described by [40]. Table 1 includes the main chemical and sensory parameters of the considered samples.

### 3.3. Volatile Emission by Electronic-Nose

**Figure 1:**
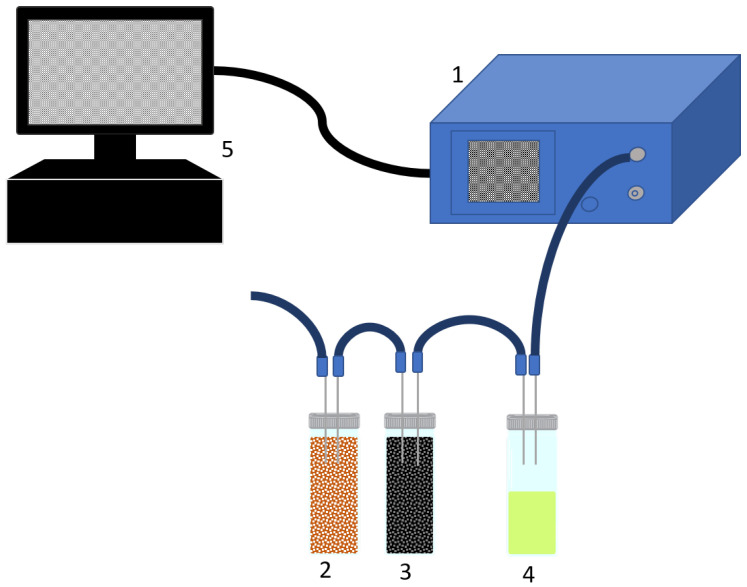
Schematic representation of the experimental setup of the electronic nose used in sample measurements (1 = PEN3 e-nose; 2 = silica gel filter; 3 = activated carbon filter; 4 = tested sample; 5 = computer).

The analysis of the headspaces of each olive oil sample was performed using an electronic nose apparatus PEN3 (Airsense Analytics GmbH, Schwerin, Germany), consisting of a gas sampling unit (maximum flow rate of 600 mL/min), an integrated sensor array composed of 10 different thermo-regulated (200–500 °C) metal oxide thick film sensors (MOS) sensitive to different classes of chemical compounds and a software (Win Muster v. 1.6.2) [41]. Figure 1 depicts a schematic of the setup used.

For each sample, 5 g of olive oil were poured in a dark glass 13.5 mL vial, hermetically sealed with rubber closure and conditioned to 30 ± 1 °C before the measurement process in order to increase the volatile concentration in the headspace.

As previously described by [41], each measurement process starts with a sensor array cleaning stage, when air, after passing through an activated carbon filter, reaches the sensor array. During the second step, air crosses another time through an activated carbon filter and then through the sample; after passing through a moisture and particle filter, air finally reaches the array of sensors. When the sample volatile compounds react with the sensing film of the sensor, an oxygen exchange occurs resulting in a change of electrical conductivity, detectable by a transducer element (electrode) attached to each sensor.

### 3.4. Data Analysis

**Figure 2:**
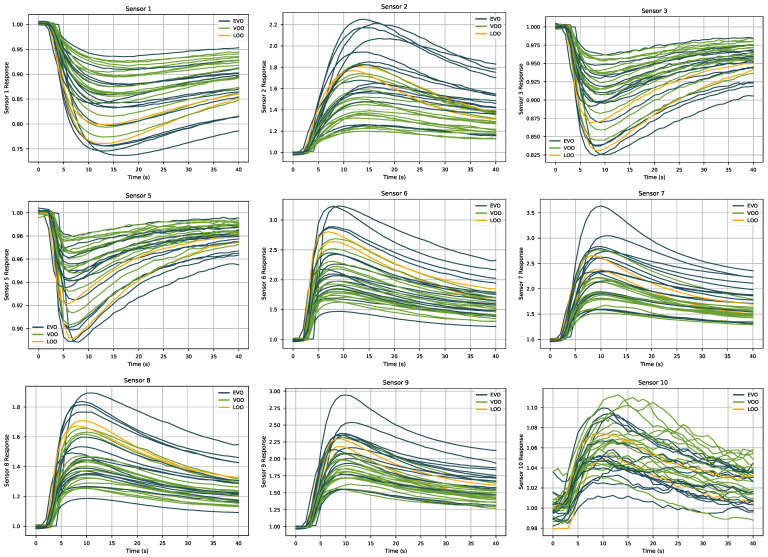
Curves of the responses of the nine sensors finally employed in the data analysis for the different samples.

The primary source of information about the samples are the response curves for each of the sensors in the PEN3 device. These curves represent the sensor measurement as a function of time. Figure 2 shows the raw response of the nine sensors finally used for the data analysis as provided by the PEN3 software for all the samples. These plots show that the noise level of the signals was very modest, so no filtering preprocessing step was required. The exception to this pattern was sensor 4 (not shown in Figure 2), which provided data that were confined in a very narrow range and with a very poor signal-to-noise ratio, so this sensor was not considered in the following data analysis.

The first step of the data analysis consisted of the extraction of features that summarized the information contained in the curves, as is common practice in applications that employ electronic noses [42]. The reason for this step is that the value at each time step is highly correlated with its neighbors, so directly using the data provided by the sensors at each time as an independent feature would grant an extremely high-dimensional feature vector with high correlations between their components, which is not desirable.

A visual inspection of the sensor response plots, along with a review of features commonly used in the literature for the characterization of this type of curves [42], provided the basis the selection of the preliminary set of features. This way, two features were extracted from the time series:

1. Peak value of the curve (SxM),
2. Final value of the curve (SxF).

This way, as an example, the peak value of the curve for sensor 1 is denoted S1M, while the final value of the curve for sensor 2 is denoted S2F. The two features extracted from the time response of each of the nine sensors finally used provide a feature vector of dimension 18, which is still relatively high when compared to the total number of samples available for the analysis (42). In order to attenuate the risk of overfitting, further regularization and dimensionality reduction techniques were applied, as detailed below. All of the computations were carried out using scikit-learn [43], a machine learning library for Python.

### 3.5. Prediction of Fruity Aroma

**Figure 3:**
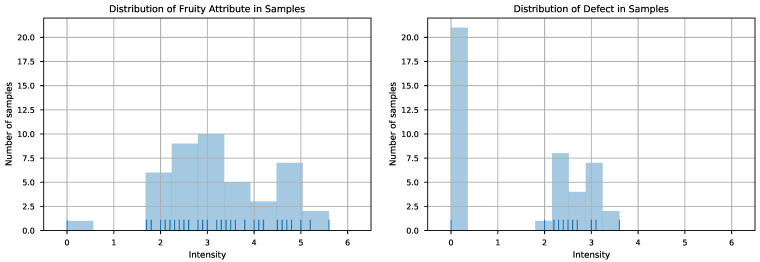
Distribution of fruity aroma and defect intensity for the studied samples.

As the value of fruity aroma is a continuous parameter, its prediction constitutes a data regression problem. The left-hand panel of Figure 3 shows the distribution of the fruity aroma values of the samples. As depicted in the plot, the values are reasonably spread, so they should not be problematic for standard regression tools. However, as commented above, the feature vector dimension was high related to the number of samples, so it was necessary to consider candidate techniques that included regularization parameters so that overfitting did not impair the generalization of the model. Furthermore, the fact that the features essentially represent the response of sensors to the concentration of volatiles makes it plausible to consider that a linear model could be appropriate for the prediction of fruity aroma. Thus, taking these considerations into account, three candidate techniques were implemented: ridge, lasso, and elastic-net regressions.

The three methods are very similar in nature, as they all essentially fit a linear model solving an optimization problem that includes some penalty in the objective function related to the size of the coefficients in the linear model, thus including regularization [44].

The ridge regression model finds the parameters *w* of the model minimizing the following expression:

$$J\left(w\right)={∥y-w^{T}x∥}^{2}+C{∥w∥}^{2}.$$

Here, *C* is the coefficient that determines the emphasis put on the regularization term. If C=0, then we would a have regular least squares fitting problem.

In turn, the lasso minimizes the following expression:

$$J\left(w\right)={∥y-w^{T}x∥}^{2}+C{∥w∥}_{1}.$$

In this case, it is the $l_{1}$ norm that penalizes the size of the parameters, thus yielding solutions that tend to be sparse, i.e., have many coefficients being exactly zero.

Finally, the elastic net is a mixture of both methods, minimizing

$$J\left(w\right)={∥y-w^{T}x∥}^{2}+C_{1}{∥w∥}_{1}+C_{2}{∥w∥}^{2},$$

with $C_{1}$ and $C_{2}$ being the parameters that determine the relative weight of the $l_{1}$ and $l_{2}$ norms, respectively.

#### Prediction of Defect

The prediction of defect required a different approach to the one used in the prediction of fruity aroma intensity. The distribution of the defect intensity values in the samples is shown in the right-hand panel of Figure 3. As depicted in the plot, many values are exactly zero—those corresponding to EVOO samples—and the rest of values are lumped between roughly 2 and 3.6. This data distribution is problematic for a regression approach due to the fact that many samples are exactly zero and the rest are concentrated in a fairly narrow range. For situations like this, it is usually best practice to start with a classification problem to determine whether the point belongs to the cluster at 0 or the other one, and then use a regression model trained exclusively with the data not being zero. However, the fact that the values are confined in a very narrow range, together with the limited number of samples available in this range, hindered the interest in training a regressor to predict these values. This way, only the classification step was pursued.

**Figure 4:**
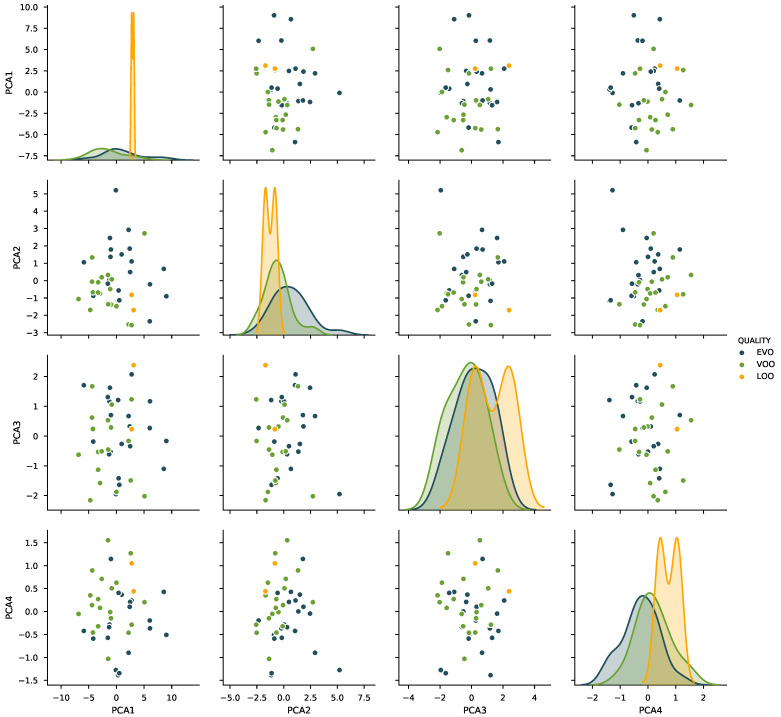
Visualization of the four first PCA components, computed for the whole dataset.

The first step in the data analysis was to perform Principal Component Analysis (PCA) to the set of 18 preliminary features, with the objective of visualizing the distribution of the classes in the feature space and obtaining an intuitive grasp of the difficulty of the task. Figure 4 shows the first four components of the PCA-transformed feature set, computed using the whole dataset available.

The inspection of the sample distribution shows that the data are effectively somewhat clustered according to its quality—which is in fact directly linked with the presence of defect—and that using a linear classifier could offer satisfactory results with good generalization properties.

This way, Support Vector Machines (SVM) were the first classifier class selected for the task. In the linearly separable case, SVM defines the separating hyperplane that maximizes the distance between the classes; for the nonlinearly separable case, as is ours, they provide an hyperplane that separates the classes including a penalty for the misclassified samples [44]. Thus, the optimization problem to be solved is:

$$\begin{array}{cccc} \; & \underset{w,b,\xi }{\mathrm{minimize}}\; & \; & \frac{1}{2}w^{T}w+C\sum_{i=1}^{n}\xi_{i}\\ \; & \mathrm{subject}\;\mathrm{to}\; & \; & y_{i}\left(w^{T}x_{i}+b\right)\geq 1-\xi_{i},\\ \; & \; & & \xi_{i}\geq 0,i=1,\cdots ,n \end{array}$$

Here, *C* is the parameter that defines the penalization of the misclassification of each sample. SVM allows for using different values of *C* for each sample, which is useful when facing an unbalanced problem, i.e., a problem where samples from one class are significantly more abundant than the other. However, we did not need to use this possibility, as roughly half of the samples are EVOO and half are not, so the value of *C* was the same for all the samples and chosen via cross-validation from a candidate set.

Another classification algorithm known to perform well for linear problems is Logistic Regression [44]. For this classifier, the optimization problem to be solved is:

$$\begin{array}{cccc} \; & \underset{w}{\mathrm{minimize}}\; & \; & \sum_{i=1}^{n}\log\left(1+e^{-y_{i}w^{T}x_{i}}\right)+C{∥w∥}^{2} \end{array}$$

Here, *C* is a regularization parameter that penalizes the size of the weight vector *w*. In this algorithm, unlike SVM, there is no explicit coefficient penalizing the misclassification of points, as it is implicit in the loss function in the term $e^{-y_{i}w^{T}x_{i}}$, which yields a large positive value whenever $w^{T}x_{i}$ and $y_{i}$ do not have the same sign.

Logistic Regression can also be performed using $l_{1}$ regularization

$$\begin{array}{cccc} \; & \underset{w}{\mathrm{minimize}}\; & \; & \sum_{i=1}^{n}\log\left(1+e^{-y_{i}w^{T}x_{i}}\right)+C{∥w∥}_{1} \end{array}$$

This formulation, analogous to the lasso regression, tends to provide a sparse *w*, which can be used to provide models using only a subset of the sensors. This approach was also tested and the results are included in the next section.

## 4. Results and Discussion

This section presents and comments on the results obtained for the prediction of fruity aroma intensity and the detection of defects.

### 4.1. Prediction of Fruity Aroma

According to the results of the sensory analysis, the fruity attribute was perceived in all the samples tested but one. Both for the prediction of fruity aroma intensity and the detection of defects, five-fold cross validation was used to test the generalization capacity of the models fitted. Within each of these first five sets, which were randomly generated from the complete set of data, four-fold cross validation was applied with the training samples to select the best parameters for the models fitted.

**Figure 5:**
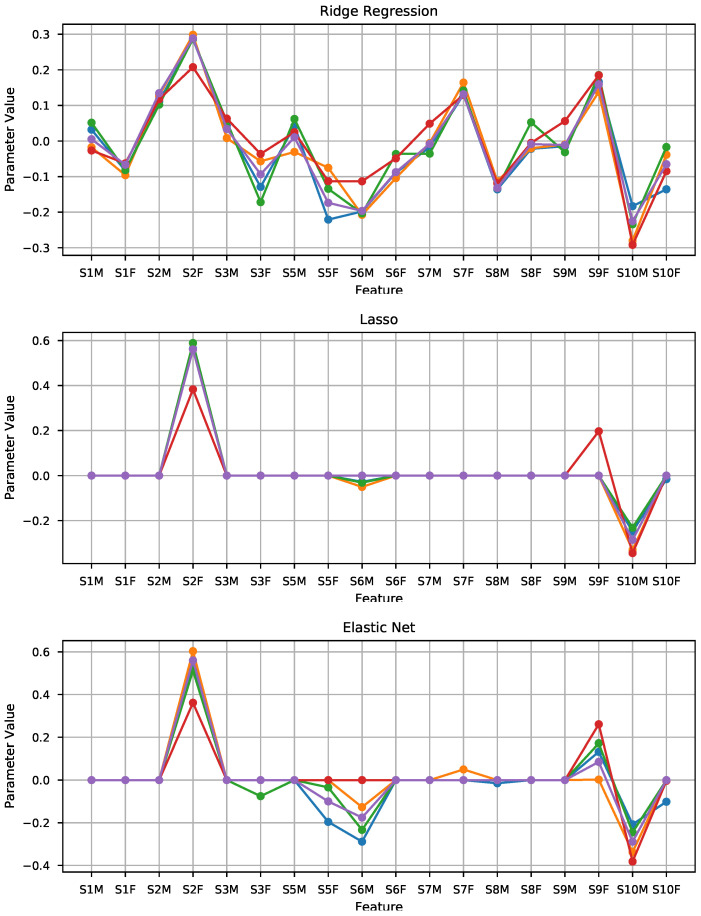
Visualization of the parameters of the lasso classifier for each of the input features.

**Figure 6:**
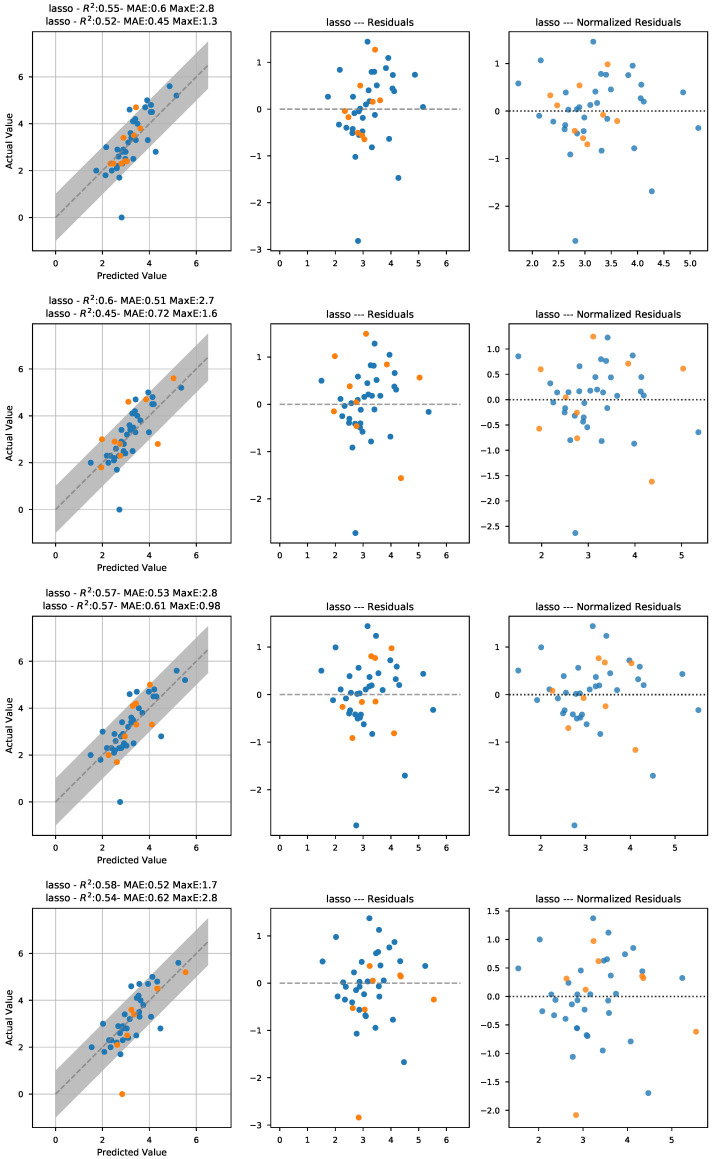
Visualization of the training and testing prediction results for the first four of the five iterations for the final models constructed using the S2F and S10M features. The gray band represents the zone of ±1. Training points are blue and testing points are orange.

As commented in the previous section, the initial approach was to fit ridge regression, lasso, and elastic net models to the 18 features presented in the previous section. Figure 5 shows the fitted parameters for each of models and the five data folds. As seen in the plot, there is a strong coherence in the values picked for all the models, with the lasso model showing the least fluctuation in the parameters found. In the lasso panel, two features clearly stand out, namely S2F and S10M. A second model using only those as input features was fitted, and the results obtained are included in Figure 6 and Table 2, together with the results of the other models.

The results obtained are fairly similar for all the models and quite satisfactory: with the final lasso model yielding a mean average error of less than 0.6 and a mean maximum error around 1.5 in the testing subsets. One final test was carried out fitting a model removing the two lampante olive oil (LOO) samples in the dataset, as an inspection of Figure 6 shows a very clear outlier corresponding to the LOO sample with 0 fruity aroma intensity. The results obtained are included in the last column of Table 2. As included in the table, the testing mean average error did not improve, but the maximum error did decrease. The most likely explanation for this point behaving as an outlier is that, in panel test analysis, it is common practice not to actually taste the olive oil if the perceived level of defect is large enough for the sample to be labeled as lampante. Thus, sometimes, there is some level of fruity aroma in the sample that might not be included in the reference results.

### 4.2. Detection of Defects

As commented in Section 3.2, the visualization of the first four components of the PCA transformation of the dataset suggested that linear classification algorithms using these transformed features should provide good results. This PCA decomposition, however, was not used for the fitting of the models.

**Figure 7:**
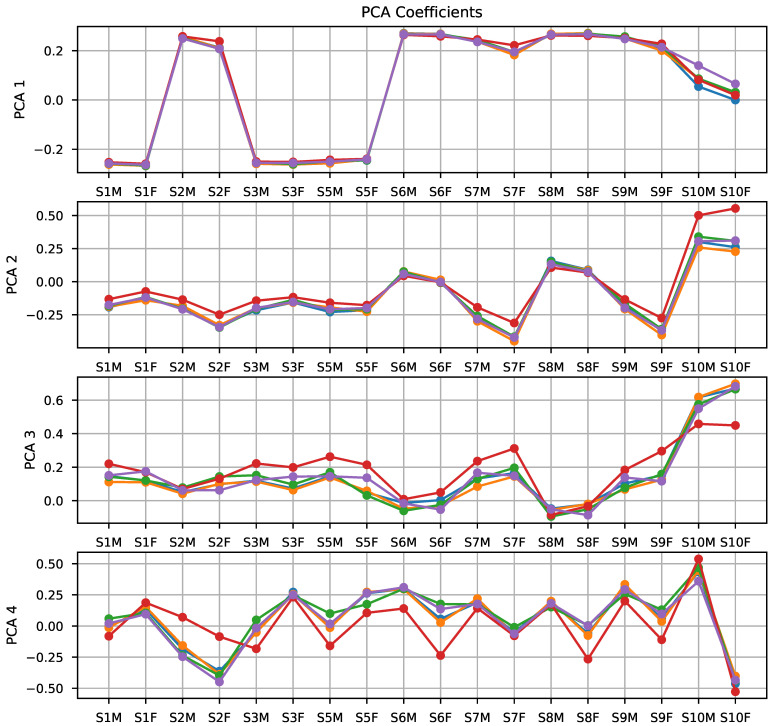
Visualization of the coefficients for the first four PCA components.

This way, the first approach was to train an SVM and a logistic regression using PCA elements in a five-fold cross-validation setup. Figure 7 shows the coefficients of the PCA transformation of each of the raw features for each fold of the cross-validation procedure. As depicted in the plot, the components provided by each subset are very similar.

A closer inspection of the discriminative component 2 (second row of Figure 7) shows that sensor 10 obtains the largest coefficient, followed by sensors 9 and 7. PCA component 4 (fourth row of Figure 7) again shows a large weight assigned to sensor 10, this time providing basically the difference between the two features associated with the sensor, as the coefficients are roughly the same magnitude and opposite sign. In this PCA component, sensor 2 also has a relatively large weight, particularly the final value feature (S2F). It is worth noting that these two sensors were the ones selected by the lasso regression for the prediction of fruity aroma intensity.

**Figure 8:**
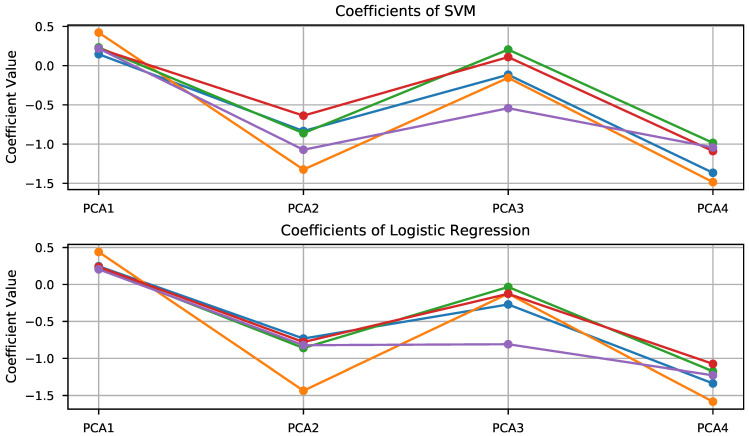
Visualization of the coefficients associated with each PCA component for the Support Vector Machine (upper panel) and Logistic Regression (lower panel).

Furthermore, Figure 8 shows the weights of the fitted models for both the SVM (top panel) and logistic regression (bottom panel). The inspection of these plots suggests that PCA components 2 and 4 are the most discriminative, as the coefficients associated with these components present a higher value than the others, particularly so for the SVM. The similarity of the coefficients yielded by both methods is noticeable, although not surprising taking into account the similarity of the optimization problems solved to obtain these parameters. The results obtained using this approach are included in Table 3. These results are not bad, but far from satisfying, particularly due to a lack of robustness shown as a large fluctuation of the classification score depending on the particular seed chosen for the random number generator responsible for the five-fold partition.

**Figure 9:**
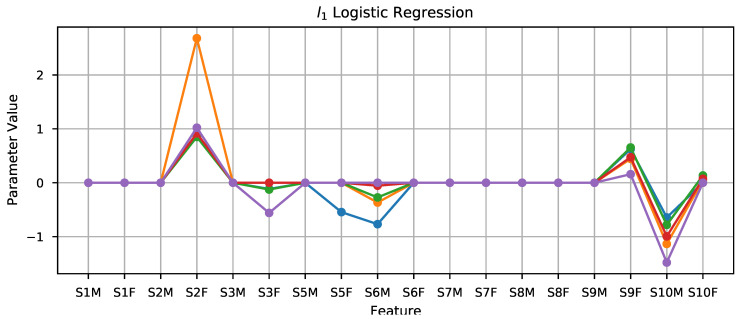
Visualization of the coefficients of the original features for the $l_{1}$ logistic regression.

**Figure 10:**
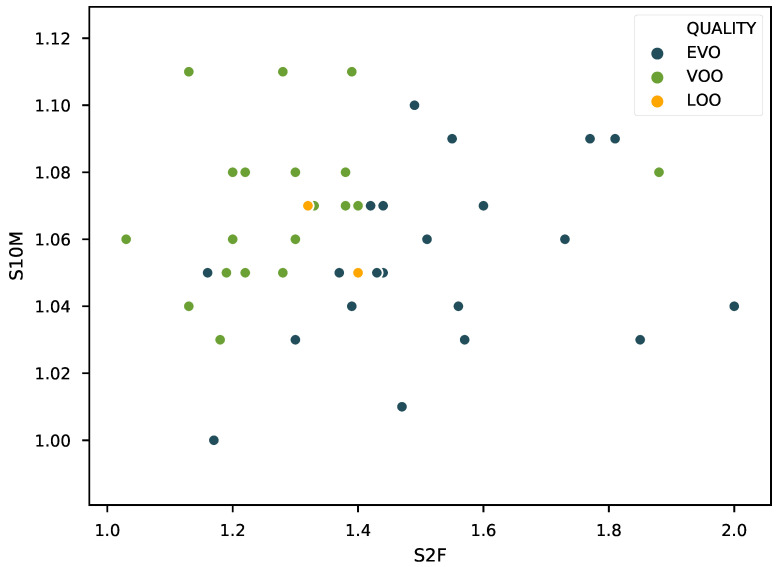
Visualization of the feature space yielded by S2F and S10M. The classes are almost completely linearly separable.

The good results obtained for the prediction of fruity aroma using the lasso motivated exploring a slightly different approach for the defect prediction problem: fitting a logistic regression with $l_{1}$ regularization using the original set of 18 features. The results obtained improved those using PCA features, yielding 89.32 and 85.56% accuracy for training and testing, respectively. Figure 9 shows the coefficients yielded by the algorithm. The inspection of this plot suggested using the same approach as with the prediction of fruity aroma, i.e., fitting a new model including only the features that got assigned weights of certain magnitude. This way, two more models were fitted, one including the features S2F, S6M, S9F and S10M; and another including just S2F and S10M. The results obtained are also included in Table 3. As seen there, the approach taking only S9F and S10M offered the best results overall (85.72% and 88.06% for training and testing, respectively) and a remarkable independence of the particular partition for the five-fold validation. The reason for these good results can be appreciated observing Figure 10. This plot shows that the samples are almost linearly separable in the space spanned by these two features. A final test was performed by fitting an SVM using just these two features, but the results obtained were poorer than those offered by the logistic regression, but are also included in the last row of Table 3.

These results are in line with the performance obtained in previous works that employed e-noses for different tasks in olive oil, such as the 91% accuracy reported to classify VOO versus other chemically treated oils [36], the 84% accuracy for the discrimination between cultivars [38], the 90% accuracy in the prediction of the olive oil quality measuring intact olives [39], and the 81% accuracy in discerning between different fruity aroma profiles [20].

## 5. Conclusions

The obtained results show that the use of electronic noses is a viable approach to perform a fast screening of the quality of produced VOO, paving the way for the development of devices capable of providing at-line or online assessments of the quality of the VOO directly in the factory. These assessments could be used to store the produced VOO in tanks with other VOO of similar quality and to apply some feedback to the process.

The plots that depict the parameters of the classifiers and regressors for the different data folds demonstrate the low variability in these values, which suggests that the classifiers and regressors are robust and should perform well when applied to new samples.

Finally, a remarkable observation is the good results obtained using $l_{1}$ regularization techniques and the comparable results obtained using only a subset of the available sensors. This is, in our opinion, very interesting for the construction of low-cost specific purpose electronic noses, as the number of sensors included in the device directly affects its total cost.

## Figures and Tables

**Table 1:** Chemical and sensory characterization of the samples (overall mean and standard deviation), and relevant quality degree, in accordance with current legislation.

Number of Samples	AI (g Oleic Acid/100 g Oil)	PV (meq O2/kg Oil)	K232	K270	ΔK	EE (mg/kg)	Fruity	Defect	Quality Grade
21	0.16 (0.03)	4.4 (0.6)	1.50 (0.10)	0.15 (0.01)	<0.01	7 (5)	4.1 (0.8)	0 (0)	EVOO
19	0.24 (0.05)	5.1 (0.8)	1.50 (0.10)	0.14 (0.01)	<0.01	8 (3)	2.4 (0.4)	2.6 (0.3)	VOO
2	0.25 (0.01)	4.3 (0.4)	1.57 (0.01)	0.17 (0.01)	<0.01	7 (1)	1.3 (1.7)	3.6 (0)	LOO

**Table 2:** Results obtained. for the prediction of fruity aroma. The final model structure selected was the fourth row (Lasso S2F, S10M).

Model	Mean Average Error (Training)	Mean Average Error (Testing)	Mean Max Error (Training)	Mean Max Error (Testing)
Ridge	1.09	1.06	5.12	3.4
ElasticNet	0.54	0.59	2.31	1.58
Lasso	0.56	0.59	2.49	1.57
Lasso (S2F, S10M)	0.55	0.55	2.55	1.49
Lasso (S2F, S10M) (VOO, EVO)	0.49	0.51	1.52	1.18

**Table 3:** Results obtained for the detection of defects. The best results are obtained using a logistic regression with $l_{1}$ using the features S2M and S10F.

Model	Training Accuracy (%)	Test Accuracy (%)
SVM PCA	85.76	80.83
Logistic Regression PCA	87.54	80.83
Logistic Regression $l_{1}$	89.32	85.56
Logistic Regression $l_{1}$ (S2, S6, S9, S10)	88.11	85.56
Logistic Regression $l_{1}$ (S2, S10)	85.72	88.06
SVM $l_{1}$ (S2, S10)	86.36	83.06

## Data Availability

The data presented in this study are available on request from the corresponding author. The data are not publicly available due to agreement terms with the company providing the samples.
